# Cross-sectional assessment of government health center needs to implement long-acting reversible contraception services in rural Rwanda

**DOI:** 10.1186/s12905-021-01555-3

**Published:** 2021-12-15

**Authors:** Amelia Mazzei, Rosine Ingabire, Etienne Karita, Jeannine Mukamuyango, Julien Nyombayire, Rachel Parker, Amanda Tichacek, Susan Allen, Kristin M. Wall

**Affiliations:** 1 Projet San Francisco, Rwanda Zambia Health Research Group, Department of Pathology and Laboratory Medicine, School of Medicine, Rollins School of Public Health, Emory University, Kigali, Rwanda; 2 Projet San Francisco, Rwanda Zambia Health Research Group, Kigali, Rwanda; 3 Rwanda Zambia Health Research Group, Department of Pathology and Laboratory Medicine, School of Medicine, Rollins School of Public Health, Emory University, 1518 Clifton Road NE, Atlanta, GA 30322 USA; 4 Department of Epidemiology, Rollins School of Public Health, Laney Graduate School, Emory University, Atlanta, GA 30322 USA

**Keywords:** Family planning, Long-acting reversible contraception, Couples, Health center needs assessment, Rwanda

## Abstract

**Background:**

There is unmet need for family planning in Rwanda. We previously developed an evidence-based couples’ family planning counseling (C)FPC program in the capital city that combines: (1) fertility goal-based family planning counseling with a focus on long-acting reversible contraceptive (LARC) for couples wishing to delay pregnancy; (2) health center capacity building for provision of LARC methods, and (3) LARC promotion by community health workers (CHW) trained in community-based provision of oral and injectable contraception. From 2015 to 2016, this service was integrated into eight government health centers in Kigali, reaching 6072 clients and resulting in 5743 LARC insertions.

**Methods:**

From May to July 2016, we conducted cross-sectional health center needs assessments in 30 rural health centers using surveys, key informant interviews, logbook extraction, and structured observations. The assessment focused on the infrastructure, materials, and human resources needed for LARC demand creation and provision.

**Results:**

Few nurses had received training in LARC insertion [41% implant, 27% intrauterine device (IUD)]. All health centers reported working with CHW, but none trained in LARC promotion. Health centers had limited numbers of IUDs (median 10), implants (median 39), functional gynecological exam tables (median 2), and lamps for viewing the cervix (median 0). Many did not have backup power supplies (40%). Most health centers reported no funding partners for family planning assistance (60%). Per national guidelines, couples’ voluntary HIV counseling and testing (CVCT) was provided at the first antenatal visit at all clinics, reaching over 80% of pregnant women and their partners. However, only 10% of health centers had integrated family planning and HIV services.

**Conclusions:**

To successfully implement (C)FPC and LARC services in rural health centers across Rwanda, material and human resource capacity for LARC provision will need to be greatly strengthened through equipment (gynecological exam tables, sterilization capacity, lamps, and backup power supplies), provider trainings and follow-up supervision, and new funding partnerships. Simultaneously, awareness of LARC methods will need to be increased among couples through education and promotion to ensure that demand and supply scale up together. The potential for integrating (C)FPC with ongoing CVCT in antenatal clinics is unique in Africa and should be pursued.

**Supplementary Information:**

The online version contains supplementary material available at 10.1186/s12905-021-01555-3.

## Background

There is considerable unmet need for family planning in Rwanda where the population density is the highest in continental Africa [1]. Family planning initiatives have the potential to improve public health via: delaying first pregnancy to improve adolescent health and gender equity [2], spacing additional pregnancies to improve maternal and child health outcomes [3], and reducing unintended pregnancy to support poverty alleviation [4].

Rwanda has achieved remarkable success in reducing its total fertility rate within the past decade from 8.6 children per woman prior to the 1994 Genocide against the Tutsi, to 6.1 children per woman in 2005, and to 4.2 children per woman in 2014–2015 [5]. However, key gaps remain including underutilization of the highly effective long-acting reversible contraception (LARC: the copper intrauterine device (IUD) and the implant). Among Rwandan women using modern contraceptive methods, only 3% use the copper IUD and 17% use the implant [6]. In rural areas, < 1.5% use the copper IUD and 15% use the implant [7].

Client-side barriers to LARC uptake, which are particularly common in rural areas, include lack of knowledge (particularly about the IUD) [6, 8–13]; lack of male involvement in family planning [8, 14–17]; concerns about side-effects; myths and misconceptions [18, 19]; and concerns about negative effects on sexual intercourse [18]. Provider-side barriers include lack of LARC knowledge and training (particularly with the IUD) [8, 9, 20, 21].

To address these barriers, researchers at Projet San Francisco (PSF) developed couples’ family planning counseling ((C)FPC). This service is a community-based family planning initiative pairing: (1) the promotion of fertility goal-based family planning that seeks to involve male partners whenever possible, (2) promotions by community health workers (CHW) as well as clinic staff, and (3) health center provision of LARC methods. In 2014, PSF began piloting (C)FPC [22] and in 2015–2016 successfully implemented the service in eight government health centers in Kigali, the capital city, reaching 6072 clients and resulting in 5743 LARC insertions [23].

At the time of research, this highly effective program had not yet been implemented outside of Kigali. Since expanding the successful (C)FPC model to rural health centers will require addressing the specific needs and barriers present in that context, a rural health center needs assessment was conducted to assess readiness to implement LARC and (C)FPC services and to identify gaps in human resources, material resources, and other areas that can be transformed into actionable steps towards equalizing access to (C)FPC and LARC methods nationwide.

## Methods

### Type of study

This project was a cross-sectional needs assessment which utilized a mixed-methods approach combining surveys, semi-structured key informant interviews using pre-identified themes, extraction of health center logbook data, and structured observations.

### Needs assessment content

Needs assessment tools were standardized and pilot tested (Additional file 1). No materials were prepared for distribution to participants. The assessment focused on the infrastructure, materials, and human resources needed for LARC demand creation and provision within the framework of family planning services, and collected data on existing service provision, current capacity and resources for scale-up of (C)FPC and LARC provision. The needs assessment collected data on: health center volume, LARC provision, human resource availability (nurses and CHW availability and training), current CHW activities within facilities and in communities, and material resource availability for LARC provision. Open-ended questions ascertained barriers to LARC or (C)FPC provision, other possible CHW duties, and funding partners for family planning.

### Site selection

In early 2016, we selected 30 rural health centers in Rwanda previously identified for potential expansion of LARC services. The health centers were distributed across 16 districts in all four non-Kigali provinces, with eleven in Eastern Province, seven in Northern Province, six in Southern Province, and six in Western Province.

### Needs assessment administration

In June 2016, Dr. Mazzei and one of two trained Rwandan PSF nurse researchers established in-person contact with the Director of each selected facility to inform them of the project and establish their buy-in prior to data collection. In-person meetings held at the respective health facility were arranged at each health center with available key staff (Director, Deputy Director, Nurse in charge of family planning, Head of CHW, data managers). Between 2 and 6 people attended each meeting per facility (meetings averaged 4 attendees). Thus, respondents were recruited by convenience to attend these meetings. No demographic data other than occupation were collected; because this was a quality improvement project, the research team did not feel that demographic data about participants was relevant. Participants responded to questions as representatives of their facilities. No one approached to attend refused, and no one was present besides the participants and researchers. No repeat interviews/focus groups were conducted in this cross-sectional project.

The needs assessment survey questions were administered to the group by a team including the first author, a student researcher, and one of two trained Rwandan PSF nurse researchers. Open-ended questions were explored in a focus group format. The team chose this interactive approach, rather than administering a written survey to each individual respondent, to encourage collaboration between health facility staff in providing thorough and accurate answers to all questions. This supported data quality by allowing for real-time discussion and consensus rather than incomplete or conflicting written responses. All interviewers/facilitators were female and had previous training and experience with conducting interviews and focus groups. Any in-group disagreements about responses were recorded and further discussed until consensus was reached, and interviewers were trained to facilitate reaching consensus. Discussions were held in a mix of Kinyarwanda, English, and French as needed according to the preferences of the assessment participants, with pauses for translation as needed and note taking. No recordings were made, and no field notes were returned to participants for feedback. Training log information and service provision data were extracted directly from health center logbooks and inventories were tallied by government clinic staff. Needs assessment data collection took an average of 2 h.

### Analysis methods

We used descriptive statistics (counts and percentages for categorical data, medians and interquartile ranges (IQRs) for continuous data) to describe health center volume, LARC provision, human resource availability (nurses and CHW availability and training), current CHW activities within facilities, and material resource availability for LARC provision. One data coder coded qualitative data using a grid with the pre-identified themes using an excel file. Findings from open-ended questions about barriers to LARC or (C)FPC provision, other possible CHW duties, and funding partners for family planning were then described using thematic qualitative data analysis methods and presented as a quantitative summary of responses.

### Ethics

All methods were carried out in accordance with relevant guidelines and regulations.

Prior to implementation of the program evaluation project in June of 2016, this program evaluation was approved by the Rwandan National Ethics Committee. Prior to implementation of the project in June of 2016, this program evaluation was determined to be non-human subjects research by the Emory Institutional Review Board criteria. The Rwandan National Ethics Committee and granted a waiver of informed consent. The intent of the project was to evaluate a specific program in Rwanda and was only meant to provide information for and about that program. The project was not designed to develop or contribute to broadly generalizable knowledge outside of the specific program evaluated. As such, no permissions/approvals were obtained from the health centers where the needs assessments took place, and no formal administrative permissions were required to access and use the programmatic data. A list of the 30 health centers from which programmatic data were collected appears at the end of this manuscript. Programmatic data was de-identified by government clinic staff before sharing with PSF investigators.

## Results

### Service integration and human resource capacity for LARC provision (Tables 1, 2)

The median health center catchment size was 37,799 people in 34 villages. Health centers inserted a median of 1 IUD, removed 0 IUDs, inserted 24 implants, and removed 4 implants in the past 3 months. Forty-three percent of the 30 facilities had integrated family planning and antiretroviral treatment, 30% had integrated family planning education in antenatal care, and only 10% had integrated family planning and HIV testing services.

Participating health centers reported a median of 13 (IQR) nurses on staff. More than half (57%) of nurses had received training in family planning service provision; even fewer had received training specific to LARC insertion (41% implant, 27% copper IUD). Almost a quarter (24%) of nurses had been trained in couples’ voluntary counseling and testing (CVCT). Health centers had a median of 2 nurses trained in CVCT, 5 in family planning, 3 in IUD insertion (and 2 actively inserting), and 4 in implant insertion (and 5 actively inserting including some who had not received formal training but had learned by observing colleagues). Participating health center staff agreed that new and/or refresher trainings were broadly needed, and 81% of nurses were viewed as potential candidates for LARC training.

**Table 1:** Human resource capacity for LARC service provision in n = 30 rural health centers, 2016

	N	%	Median per health center	IQR
*Health center volume and LARC provision*				
Catchment population of health centers	931,873	–	27,799	18,340
Villages in health center catchment areas	1019	–	33	13
IUD insertions in last 3 months	73	–	1	2
IUD removals in last 3 months	9	–	0	0
Implant insertions in last 3 months	1019	–	24	33
Implant removals/replacements in last 3 months	219	–	4	7
*Service integration*				
Services integrated with FP				
FP and antiretroviral treatment	13	43%	–	–
FP and antenatal care	9	30%	–	–
FP and HIV testing	3	10%	–	–
*Human resources—nurses*				
Nurses	414	–	13	5
Nurses trained in:				
CVCT provision	100	24%	2	3
FP provision	234	57%	5	10
IUD insertion	113	27%	3	4
Implant insertion	170	41%	4	4
Nurses actively inserting				
IUD	72	17%	2	2
Implant	185	45%	5	4
Health centers needing nurse refresher trainings needed in FP, IUD, and implant	30	100%	30	0
Nurses in health center that could be trained to insert LARC	337	81%	12	5
*Human resources—CHW*				
CHW working with all health centers	3071	–	101	39
Villages with CHW trained in FP	955	94%	30	12
CHW formally trained in LARC promotion	0	0%	0.0	0.0
*Clinics with CHW FP activities*				
Dispensing oral contraceptive pills				
CHW already conducting	30	100%	–	–
*Administering Depo-Provera injections*				
CHW already conducting	30	100%	–	–
*Providing LARC education and promotion*				
CHW already conducting	3	10%	–	–
This could be done by CHW	27	90%	–	–

*CVCT* couples’ voluntary HIV counseling and testing, *LARC* long-acting reversible contraceptive, *FP* family planning, *IUD* intra-uterine device, *IQR* interquartile range, *CHW* Community Health Worker

**Table 2:** Descriptive details of participating facilities, 2016

Health center name	District	Catchment population	# villages served	# social workers	# nurses	# community health workers
*Northern Province*						
Muhoza (Ruhengeri) HC	Musanze	73,563	49	2	26	147
Karwasa HC	Musanze	31,752	30	1	11	90
Tare HC	Rulindo	20,755	35	2	11	105
Bushoka HC	Gakenke	18,681	28	2	11	84
Cyabingo HC	Gakenke	17,684	34	0	8	102
Gitare HC	Burera	20,418	33	1	13	113
Gahunga CS	Burera	24,319	39	1	8	117
*Eastern Province*						
Remera HC	Ngoma	29,898	33	1	10	99
Kibungo HC	Ngoma	59,618	59	1	13	177
Nyamata HC	Bugesera	39,095	47	0	15	141
Mayange HC	Bugesera	32,697	35	2	17	105
Rugarama (Gatsibo) HC	Gatsibo	45,219	58	1	13	174
Kabarore HC	Gatsibo	40,577	24	2	17	72
Mukarange HC	Kayonza	46,375	30	0	15	90
Gahini HC	Kayonza	39,990	35	1	11	105
Rwamagana HC	Rwamagana	51,416	52	2	20	156
Nyagasambu HC	Rwamagana	26,126	33	2	14	99
Mwogo HC	Bugesera	19,666	25	1	11	75
*Southern Province*						
Kigeme HC	Nyamagabe	21,395	8	4	13	24
Kigoma HC	Ruhango	27,328	48	1	9	144
Nyanza HC	Nyanza	28,030	35	1	14	105
Nyamagabe HC	Nyamagabe	19,444	21	3	16	63
Kamonyi HC	Kamonyi	27,568	23	1	16	69
Musambira HC	Kamonyi	33,954	31	4	16	93
*Western Province*						
Kibogora HC	Nyamasheke	21,798	26	2	13	78
Giheke HC	Rusizi	21,094	36	0	8	108
Nyamasheke HC	Nyamasheke	36,392	50	1	18	150
Gihundwe HC	Rusizi	29,279	31	1	16	93
Bigogwe Surg. Med. Center	Nyabihu	16,007	18	2	18	54
Kora HC	Nyabihu	11,735	13	1	13	39

All health centers reported working with CHW (median of 101 CHWs/health center). The number of CHW depended on the number of villages within the health center’s catchment area. Each health center reported having 3 CHW per village: typically, one focused on maternal and child health while the other two attended to all other areas including family planning. Most (94%) health centers reported that every village they serve had at least one CHW trained in family planning service provision (8 health centers were in the process of training CHW in family planning). At the time of the assessment, 64 villages (6%) were without a family planning-trained CHW. None of the health centers reported that their CHWs were trained in LARC promotion.

CHW charged with family planning were involved in community-based provision (CBP) of oral contraceptive pills (OCPs) and Depo-Provera injections (100% were performing these activities) and providing LARC education (10% actively providing though without formal training, with the remainder able to provide if trained).

### Material resources for LARC service provision (Table 3)

Health centers generally had a limited number of IUDs in stock (median 10) and relatively more implants (median 39). All health centers reported that they could procure additional IUDs and implants from their local district pharmacy as needed (although some reported occasional stock-outs that could persist for months). The most notable issues with availability of materials and equipment were those affecting IUD provision and included a lack of functional gynecological exam tables available for family planning use (median 2/health center) and lamps for viewing the cervix (median 0/health center). Many health centers reported sharing exam tables with other departments and some reported improvising using cellphone lights or flashlights to illuminate the cervix for IUD insertion or the skin for implant insertion. All health centers had access to either an autoclave or dry heat sterilization oven. However, not all heat sterilization devices were able to accommodate the size of insertion kit components (particularly the tenaculum and uterine sound), and some sterilization devices required more power than was available through the health center’s power supply. Health centers that did not use their own sterilization devices had arrangements with local hospitals anywhere from 0 to 7 km away that allowed them to sterilize their equipment. When asked how many insertion kits could be sterilized in a day, responses ranged from 0 to 10 (median 3/day). Most health centers had access to promotional materials for LARC (87%). Half of participating health centers had access to a functional TV and media player of some kind. All participating health centers reported having electricity, though only 60% had functional backup power supplies. All health centers reported that they had the following on-site: materials and antiseptics to clean the cervix for IUD insertion, sterile gloves, local anesthetic, materials and antiseptics to clean the arm for implant insertion, and bandages for the arm.

**Table 3:** Material resources available for LARC service provision in n = 30 rural health centers, 2016

	Median	IQR
*Median number of:*		
IUD methods	10	9
Hysterometer	3	3
Lamp for viewing cervix	1	1
Forceps for IUD removal	0	0
Tenaculum	3	3
Speculum	5	7
Gynecological table	2	2
Implant methods	39	71
Disposable implant kit	39	50
Reusable implant kit	0	5
Halogen lamp	0	0
Scalpel	91	119

	N	%
*Clinics with availability of:*		
Autoclave or dry heat sterilization oven	30	100
Electricity	30	100
Backup power supply	18	60
Materials and antiseptics for IUD insertion	30	100
Sterile gloves	30	100
Local anesthetic	30	100
Materials and antiseptic to clean the arm	30	100
Bandages for the arm	30	100
Promotional materials for the IUD or implant	26	87
Television and media player	15	50

*LARC* long-acting reversible contraceptive, *IUD* intrauterine device, *IQR* interquartile range

### Open-ended stakeholder questions regarding LARC service provision (Table 4)

Health centers anticipated obstacles to increase LARC services including the need to train nurses (87% of health centers), low client acceptability (87%), and the need for more equipment (50%). Health centers anticipated relatively fewer obstacles to implementing (C)FPC programming, though 77% cited that it will be difficult to engage men in couples-based family planning. The reported reasons for this anticipated obstacle (not tabled) included cultural norms (family planning is generally seen as a woman’s responsibility), gendered behavior (men are not willing to wait in line at the health center as women are), poverty (attending as a couple doubles the transportation cost and means that the male partner will be missing opportunities for earned income), and fact that men may live in separate towns due to work obligations. Staff in 16 of the health centers felt that CHW were overloaded and could not take on additional duties. Health center staff consistently emphasized the fact that CHW were overworked and undercompensated; in some cases, CHW were incurring expenses as they were not being compensated for their transportation, use of their home for health activities, and personal materials. The majority of health centers stated that they had no funding partners for family planning assistance (60%), with 30% reporting support from USAID.

**Table 4:** Open-ended questions about LARC service provision in n = 30 rural health centers, 2016

	N	%
*Barriers to introduce or expand LARC services?*		
Need for nurse training	26	87
Low client acceptability^a^	26	87
Need for more equipment	15	50
Cost barriers for uninsured	13	43
Need for CHW promotional training	7	23
Understaffing	4	13
None	1	3
*Obstacles in implementing (C)FPC?*		
Improving male involvement	23	77
None	5	17
Cost barriers for uninsured	3	10
Client acceptability^a^	2	7
Need for CHW training	2	7
Understaffing/wait times	2	7
Obtaining provider buy-in	1	3
*Partners/funders that help provide family planning services?*		
Maternal child survival program (USAID)	9	30
Partners in health	1	3
Ministry of health	1	3
Global fund	1	3
None	18	60

*LARC* long-acting reversible contraceptive, *FP* family planning, *IUD* intra-uterine device, *CHW* community health worker

^a^Client acceptability: myths/misconceptions/rumors, lack of awareness particularly among men, concerns about side-effects or adverse events, religious beliefs

## Discussion

LARC provision and uptake, especially for the IUD, is low at rural health centers despite the availability of LARC methods and LARC insertion trainings for rural providers in some areas [24]. Needs assessment findings emphasized the need for: (1) LARC equipment, (2) LARC insertion trainings for nurses with opportunities to practice these skills regularly, (3) LARC promotions in the clinic and by CHW involving male partners when possible, (4) Family planning integration with other services including HIV, and (5) funding partners.

### Equipment

Several health centers needed functional gynecological exam tables and lamps designated for family planning department use only in order to scale-up IUD provision. On-site access to adequately sized and functional sterilization devices, along with a sufficient power supply and backup power to run them, are essential components.

### LARC training

The proportion of nurses in each health center who actively insert LARC methods was low (17% IUD, 45% implant). All nurses should be trained/re-trained in family planning service provision, including IUD and implant insertion and removal. Nurses often informally train each other on implant insertion and quickly become confident with this relatively straightforward procedure. In contrast, IUD insertions require more technical skill and confidence is quickly lost if these skills are not maintained through regular practice. These findings are similar to a 2014 rapid assessment of Zambian family planning clinics which found that, after LARC training, the proportion of nurses who were inserting Jadelle was much higher (96%) than IUDs (30%) [25]. A study in South Africa and Zimbabwe found that provider misconceptions about the IUD persisted after training (for example, < 5% reported that IUDs were appropriate for women with or at high risk for HIV), but that clinicians and nurses, especially in rural area, were eager to be trained/retrained on the IUD [26].

### CHW promotions

Improving LARC—especially IUD—services must be accompanied by increases in demand so that providers are able to practice and maintain their skills. Rural health centers have robust networks of CHW who are able to reach those in the community who may not be attending the health center, including OCP and Depo-Provera users [27]. We found that no CHW were formally trained to promote LARC methods and only three health centers reported that CHW were doing so. The effectiveness of CHW in promoting (C)FPC and LARC services has been demonstrated in Kigali [22, 23] as well as across 14 countries in a Marie Stopes International implementation of LARC services [28], and is likely to be transferrable to the rural context in Rwanda.

All associated CHW should be trained in promotion of (C)FPC and LARC to dispel myths which are common in rural areas [8, 9]. As CHW are often trusted individuals who are close to the community and hold some influence [29], they are well-positioned to lead these promotional efforts. Additionally, as CHW visit homes they are able to increase male involvement in family planning decisions, a critical component of successful LARC promotion in other studies [8, 14]. Focus groups conducted in Rwandan CHWs indicated that challenges to delivery of health care services included overwhelming workload, insufficient trainings, and poor supervision. CHW are not civil servants and their remuneration depends on a co-operative system with various sources of revenue. CHW reported that while money was an important incentive, they were also motivated by community value and respect [29].

### In-clinic promotions

(C)FPC and LARC promotion can take place in the health center as well as through CHW in the community; opportunities for in-clinic promotions exist within infant vaccination, HIV testing, outpatient and antiretroviral treatment services. Rwanda is the only country in Africa to have offer CVCT as nationwide standard of care at the first antenatal visit [30]. Health centers may be able to leverage the presence of male partners at CVCT services by offering add-on (C)FPC in the same session. Studies in Rwanda and Zambia have shown that knowledge of LARC methods is poor among men [31], and that fertility-goal based family planning provided to couples when access to LARC is ensured increases uptake of both IUDs and implants [22, 32, 33]. More recently, post-partum IUD (PPIUD) insertion has been feasible and acceptable in Kigali [34, 35]. Promotions for PPIUD would ideally take place prior to labor and involve male partners. PPIUD was not addressed during this needs assessment but if services were made available, this LARC option could also be discussed during (C)FPC at the first antenatal visit. Half of participating health centers had access to a functional TV and a media player of some kind. This can be leveraged to develop and deliver recorded education about LARC suitable for illiterate clients, as well as visual illustrations of LARC insertions [36].

### HIV/family planning integration

Integrating family planning and HIV services has been a major goal of international stakeholders [37, 38] to reduce unintended pregnancy and perinatal HIV transmission [39]. Integrating family planning (including LARC) and HIV services is a health policy priority in Rwanda [40, 41]. However, current policies have not yet resulted in integration nor nationwide promotion of LARC methods [40, 41]. Data from a recent qualitative study of interviews with key Rwandan policymakers and stakeholders indicated that the best way to integrate HIV and family planning services was through development of integrated training materials, data collection tools, and advocacy and policy guidance [42].

### Partners and funders

The health centers had limited financial support for family planning and few non-governmental partnerships. Further advocacy with stakeholders is critical.

Maintaining adequate stocks of LARC methods and related insertion supplies is necessary for increased LARC provision but is not sufficient without the presence of functional sterilization equipment and a reliable power supply. The choice of disposable versus non-disposable implant insertion kits should be matched to the health center’s capacity for reliable and timely equipment sterilization. Disposable insertion kits are convenient but comparatively expensive and wasteful when compared with reusable insertion equipment. Many health centers already have stocks of reusable specula and scalpels that can be used with the proper sterilization equipment. Strategies to enhance nurses’ skill at inserting IUDs are needed including overcoming misconceptions that may persist after training as well as ongoing supervision and feedback regarding IUD insertion. Additional training of rural CHW will be required, and a key barrier is the high existing workload of CHW. Use of educational tapes/DVDs in health center waiting rooms may be an effective way to promote family planning services including LARC methods. Funding partnerships to support the purchase of dedicated, functional, durable family planning equipment; the installation of reliable backup power sources adequate to provide electricity for sterilization machines; the development and implementation of skills-based LARC insertion trainings and promotional materials for health centers; and training and compensation for CHW LARC promoters are urgently needed.

Future research will need to identify funding partnerships to support resource capacity for LARC provision (including equipment, provider trainings and follow-up supervision, and community awareness). Studies are needed to adapt existing materials developed for (C)FPC training and promotion in Kigali for the rural context.

This study is a comprehensive rural government health center needs assessment related to LARC services in Rwanda. Limitations include possible social desirability bias leading respondents to understate their need, which we attempted to mitigate by describing to the participants that none of the findings would be expressly linked to a given health center and that no future resources would be limited based on performance. Alternately, a desire to maximize the likelihood of future support could have had led to an overstatement of need. With these possible sources of bias in mind, health center staff comments regarding capacity for LARC insertion were triangulated with monthly LARC provision data from health center logbooks to validate qualitative data on capacity gaps. Similarly, responses about available materials, equipment, and infrastructure were paired with structured observations in each facility to confirm staff accounts whenever possible. These data were mutually validating. Finally, these findings are not meant to be generalizable outside of the Rwandan health centers which our program were evaluating.

## Conclusions

To successfully implement LARC and (C)FPC services in rural health centers, material and human resource capacity for LARC provision will need to be greatly strengthened through equipment, provider trainings and follow-up supervision (especially for the IUD), and supporting funding partnerships. Simultaneously, community awareness of LARC methods among women and their male partners must be increased through community-based and clinic-based education and promotion to ensure that promotion and provision of LARC scale-up together.

## Health centers from which programmatic data were collected

**Table:** 

Bigogwe Surgical Medical Center
Bushoka HC
Cyabingo HC
Gahini HC
Gahunga CS
Giheke HC
Gihundwe HC
Gitare HC
Kabarore HC
Kamonyi HC
Karwasa HC
Kibogora HC
Kibungo HC
Kigeme HC
Kigoma HC
Kora HC
Mayange HC
Muhoza (Ruhengeri) HC
Mukarange HC
Musambira HC
Mwogo HC
Nyagasambu HC
Nyamagabe HC
Nyamasheke HC
Nyamata HC
Nyanza HC
Remera HC
Rugarama (Gatsibo) HC
Rwamagana HC
Tare HC

## Supplementary Information

**Additional file 1**. Clinic Needs Assessment.

## Data Availability

The datasets used and/or analyzed during the current study are available from the corresponding author on reasonable request.

## Abbreviations

LARC: Long-acting reversible contraception
IUD: Intrauterine device
(C)FPC: Couples’ family planning counseling
CHW: Community health workers
CVCT: Couples’ voluntary counseling and testing
CBP: Community-based provision
OCPs: Oral contraceptive pills
PPIUD: Post-partum IUD
IQR: Interquartile range
