# Supplementary material for: Cross-sectional assessment of government health center needs to implement long-acting reversible contraception services in rural Rwanda
Source: BMC Womens Health. 2021 Dec 15;21:411. doi: 10.1186/s12905-021-01555-3 (PMC8672509; doi:10.1186/s12905-021-01555-3)
Supplement: Supplementary file 1 — Additional file 1. Clinic Needs Assessment. [file 12905_2021_1555_MOESM1_ESM.docx]

**CLINIC NEEDS ASSESSMENT**

Province (PROVINCE) _______________________________________________________________________________________________________

District (DISTRICT) _____________________________________________________________________________________________________________

Health Center (CLINIC) ________________________________________________________________________________________________________

PSF Data Collector Initials (Dataint) _____________Date (Day/Month/Year): **___ ___ /___ ___ ___ /___ ___**

Interviewee Name, First/Last (CNAee) _______________________________________________________________________________________

Position (CNAeePos)________________________________________________________________________________________________ ___________

Contact Number (CNAeeNum)_________________________________________________________________________________________________

1. Name and contact number of the Titular of your health center (*if NOT the interviewee*):

**Nom et contact de Titulaire du centre de santé :**

Name **Nom** (Titularname)­­­­­­­­ ________________________________________________________________________________________________

Contact Number **Numero de Contact** (CICNum) ______________________________________________________________________

1. Please estimate the catchment population of your health center **Population servi par le centre de santé** (CatchP) ___________________________________
2. Is clinic urban or rural**? Le centre de santé est-il urbain ou rurale?** (Clintype)

- Urban (1)
- Rural (2)

1. Is clinic Catholic? **Le centre de santé est-il un centre catholique?** (Catholic)

- Yes (1)
- No (2)

1. Please identify clinic activities that occur during the weekdays (clinicschedule)

**Veuillez identifier les activités du centre qui se produisent au cours de la semaine :**

|  | **Monday**  **Lundi** | **Tuesday**  **Mardi** | **Wednesday**  **Mercredi** | **Thursday**  **Jeudi** | **Friday**  **Vendredi** |
| --- | --- | --- | --- | --- | --- |
| AM | - Family planning  - clinic - LARC (IUD/Implant) offering - ANC first - ANC subsequent - Under 5 first /post-natal first - Under 5 subsequent - ART - VCT - CVCT - PMTCT - OPD - Nutrition - PIT - Other _______ - Other _______ | - Family planning  - clinic - LARC (IUD/Implant) offering - ANC first - ANC subsequent - Under 5 first /post-natal first - Under 5 subsequent - ART - VCT - CVCT - PMTCT - OPD - Nutrition - PIT - Other _______ - Other _______ | - Family planning  - clinic - LARC (IUD/Implant) offering - ANC first - ANC subsequent - Under 5 first /post-natal first - Under 5 subsequent - ART - VCT - CVCT - PMTCT - OPD - Nutrition - PIT - Other _______ - Other _______ | - Family planning  - clinic - LARC (IUD/Implant) offering - ANC first - ANC subsequent - Under 5 first /post-natal first - Under 5 subsequent - ART - VCT - CVCT - PMTCT - OPD - Nutrition - PIT - Other _______ - Other _______ | - Family planning  - clinic - LARC (IUD/Implant) offering - ANC first - ANC subsequent - Under 5 first /post-natal first - Under 5 subsequent - ART - VCT - CVCT - PMTCT - OPD - Nutrition - PIT - Other _______ - Other _______ |
| PM | - Family planning  - clinic - LARC (IUD/Implant) offering - ANC first - ANC subsequent - Under 5 first /post-natal first - Under 5 subsequent - ART - VCT - CVCT - PMTCT - OPD - Nutrition - PIT - Other _______ - Other _______ | - Family planning  - clinic - LARC (IUD/Implant) offering - ANC first - ANC subsequent - Under 5 first /post-natal first - Under 5 subsequent - ART - VCT - CVCT - PMTCT - OPD - Nutrition - PIT - Other _______ - Other _______ | - Family planning  - clinic - LARC (IUD/Implant) offering - ANC first - ANC subsequent - Under 5 first /post-natal first - Under 5 subsequent - ART - VCT - CVCT - PMTCT - OPD - Nutrition - PIT - Other _______ - Other _______ | - Family planning  - clinic - LARC (IUD/Implant) offering - ANC first - ANC subsequent - Under 5 first /post-natal first - Under 5 subsequent - ART - VCT - CVCT - PMTCT - OPD - Nutrition - PIT - Other _______ - Other _______ | - Family planning  - clinic - LARC (IUD/Implant) offering - ANC first - ANC subsequent - Under 5 first /post-natal first - Under 5 subsequent - ART - VCT - CVCT - PMTCT - OPD - Nutrition - PIT - Other _______ - Other _______ |

1. We are interested in finding out which services are integrated at this clinic. For each of the services listed above, please identify if they are integrated with the activities in another department, and if so, how they are integrated. (Integrated)

**Nous sommes intéressés à savoir quels services sont intégrés au centre de santé. Pour chacun des services énumérés ci-dessus, veuillez identifier ceux qui sont intégrés aux activités dans un autre département et comment ils sont intégrés**

______________________________________________________________________________________________________________________________

______________________________________________________________________________________________________________________________

______________________________________________________________________________________________________________________________

1. If Catholic clinic, is there a health post nearby that provides family planning services? (HealthPost)

**Si le centre de santé est catholique, ya-t-il un poste de sante tout près qui fournit les services de la planification familiale ?**

- - Yes (1)
  - No (2)
  - Not applicable - **clinic not Catholic (Skip to Question 11)** (3)

1. What is/are the name of the nearest health post? **Quel est le nom du poste de santé tout près?** (NameHP) ___________________________________________________________________________________________
2. How many minutes does it take to walk from the clinic to the nearest health post? **Combien des minutes prend-t-il pour marcher du centre de santé catholique au poste de santé le plus proche?** (MinutesHP)_________________
3. If a Catholic health center, do you refer to the nearest health post? **Si votre centre de santé est catholique, est-ce que vous referez les clients au poste de santé pour les services que vous ne fournissez pas (c’est-à-dire la PF etc.)?** (RefHP)
   - Yes (1)
   - No (2)

**THIS IS THE LAST QUESTION FOR ALL CATHOLIC CLINICS.**

1. How many social workers and nurses are there in your health center? How many are trained in the following services? **Combien des assistants sociale et infirmières avez-vous à votre centre de santé? Combien parmi eux sont formés dans les services suivants ?**

|  | Total Employees | Trained in CVCT | Trained in DATA | Trained in FP | Trained in LARC: IUD | Trained in LARC: Implant |
| --- | --- | --- | --- | --- | --- | --- |
| Social Workers | (swttl) | (swcvct) | (swdata) | (swfl) | (swiud) | (swimp) |
| Nurses | (nursttl) | (nurscvct) | (nursdata) | (nursfp) | (nursiud) | (nursimp) |

1. Who at the clinic is typically responsible for compiling the Family planning reports which are sent to the MOH? **Qui au centre de santé est responsable de la compilation des rapports de la PF qui sont envoyé au MOH?** (FPrepMOH)
   - Clinic Titular/In charge (1)
   - Family Planning Supervisor (2)
   - Data Manager (3)
   - Other (4) Specify (FPreportother): __________________________________________________________________________
2. Who at the clinic is typically responsible for compiling the HIV reports which are sent to RBC? **Qui au centre de santé est responsable de la compilation des rapports de VIH qui sont envoyé au RBC?** (HIVrepRBC)
   - Clinic Titular/In charge (1)
   - HIV Supervisor (2)
   - Data Manager (3)
   - Other (4)
3. If ‘Other’, please specify: (RepotOther)__________________________________________________________________________________
4. Please provide the name and position of the person in charge of ordering the following supplies at your clinic **Veuillez fournir le nom et poste de la personne chargée de commander les équipements et fournitures suivants**:
5. Family Planning Methods **Méthodes PF** – IUD/Implant

Name **Nom**(LARCmethname):___________________________________________________

Position **Poste**(LARCmethpos): _________________________________________________

How often **A quelle fréquence?** (monthly, yearly etc)? (LARCmethFreq)_______________________________________________

1. Laboratory supplies **Fournitures de laboratoire**

Name **Nom**(SupLabname):___________________________________________________

Position **Poste**(Suplabpos): _________________________________________________

How often? **A quelle fréquence** ?(monthly, yearly etc) (SupLabFreq) ________________________________________________________________

1. Pharmacy supplies **Fourniture de pharmacie**

Name **Nom**(SupPharmname):___________________________________________________

Position **Poste**(SupPharmpos): _________________________________________________

How often? **A quelle fréquence** (monthly, yearly etc) (SupPharmFreq) ____________________________________________________________

1. HIV Test Kits **Kits des tests VIH**

Name **Nom**(HIVkitname):___________________________________________________

Position **Poste** (HIVkitpos): _________________________________________________

How often? **A quelle fréquence** (monthly, yearly etc) (HIVKitFreq)_________________________________________________________________

1. Clinic supplies for IUD/Implant such as speculum, tenaculum, gynaecological tables, etc.

**Les fournitures cliniques pour DIU / implants y compris spéculum, tenaculum, tables gynécologiques, etc**

Name **Nom**(SupLARCname):___________________________________________________

Position **Poste**(SupLARCpos): _________________________________________________

How often? **A quelle fréquence** (monthly, yearly etc) (SupLARCFreq)______________________________________________________________

1. Can you procure IUDs and Implants through the district pharmacy/CAMERWA if we provide LARC training (Procpharm)? **Pouvez-vous obtenir les DIU et les implants à travers la pharmacie de district et/ou CAMERWA si nous offrons une formation sur les méthodes de longues durées**
   - Yes (1)
   - No (2)
2. Does your clinic have a reliable supply of electricity? (ClinicElec) **Votre centre de santé a-t-il un approvisionnement fiable en électricité ?**
   - Yes (1)
   - No (2)
3. Does your clinic have a generator? (Clinicgen) **Votre centre de santé a-t-il un générateur ?**
   - Yes (1)
   - No (2)

18b. If Yes, does it function (Genfunc)? Specify: **Si oui, fonctionne-t-il ? Specifiez :** _____________________________________________________________________________________________________________________________

1. Does your clinic have the following audio visual equipment? **Votre centre de santé a-t-il les équipements audio-visuels suivants ?**
2. TV (ClinicTV)
   - Yes (1)
   - No (2)
3. VCR (ClinicVCR)
   - Yes (1)
   - No (2)
4. DVD player (ClinicDVD)
   - Yes (1)
   - No (2)

19d. If yes to any above, do they function (videquipfunc)? Specify: **Si oui pour les questions ci-dessus, fonctionnent-ils? Spécifiez :** __________________________________________________________________________________________________________________

1. Names and phone numbers for people involved in CVCT or family planning program at your clinic? (e.g. nurses, titulaires, FP in-charges)

**Veuillez fournir les noms et numéros de contacts pour le personnel qui est affilié avec les services de PF et CVCT au centre de santé**

| Name: (N1Name) | Position: (N1Position) | Contact: (N1Contact) |
| --- | --- | --- |
| Name: (N2Name) | Position: (N2Position) | Contact: (N2Contact) |
| Name: (N3 Name) | Position: (N3Position) | Contact: (N3Contact) |
| Name: (N4 Name) | Position: (N4Position) | Contact: (N4Contact) |
| Name: (N5Name) | Position: (N5Position) | Contact: (N5Contact) |
| Name: (N6Name) | Position: (N6Position) | Contact: (N6Contact) |
| Name: (N7Name) | Position: (N7Position) | Contact: (N7Contact) |
| Name: (N8Name) | Position: (N8Position) | Contact: (N8Contact) |
| Name: (N9Name) | Position: (N9Position) | Contact: (N9Contact) |
| Name: (N10Name) | Position: (N10Position) | Contact: (N10Contact) |
| Name: (N11Name) | Position: (N11Position) | Contact: (N11Contact) |
| Name: (N12Name) | Position: (N12Position) | Contact: (N12Contact) |

1. How many nurses/other staff in your health center insert LARC (implant or IUDs)? (LARCStaff)

**Combien des infirmières au centre de santé peuvent insérer les méthodes de longues durées (DIU/Jadelle)** _________________

1. How many nurses/other staff in your health center can be trained to insert LARC (implant or IUDs)? (nurstrn)

**Combien des infirmières au centre de santé peuvent être formées pour insérer les méthodes de longues durées (DIU/Jadelle)** _________________

1. How many IUD insertions were performed in your clinic in the last three months? (IUDnum)

**Combien des DIUs étaient inséré au centre de santé pendant les dernières 3 mois ?**____________________

1. How many IUD removals were performed in your clinic in the last three months? (IUDrem)

**Combien des DIU étaient enlevé au centre de santé pendant les dernières 3 mois ?** _______________

1. How many implant insertions were performed here in the last three months? (IMPNum)

**Combien des implants étaient inséré au centre de santé pendant les dernières 3 mois ?** ______________

1. How many implant removals were performed here in the last three months? (IMPrem)

**Combien des implants étaient enlevé au centre de santé pendant les dernières 3 mois** ?__________________

1. How many items for insertion of IUD do you have in your clinic? **Votre centre de santé a-t-il combien des items pour l’insertion de DIU ?**

Number of IUDs **Nombre de DIU?** (IUDmeth) ______

Number of forceps **Nombre de forceps?** (IUDFor) ________
Autoclave **Autoclave**(IUDAuto) Yes (1) No (2)

- Are you using the autoclave to sterilize LARC equipment **Est-ce que vous utilisez l’autoclave pour stériliser l’équipement des méthodes de longues durées** (autoLARC) Yes (1) No (2)
- How many LARC insertion kits (IUD/Implant) can be sterilized in a day (autoKitnum)?

**Combien des kits d’insertion des méthodes de longues durées peuvent être stérilisés au cours d’une journée ?** ______

Number of Speculum **Nombre de speculums** (IUDSpec) ________
Number of Hysterometer **Nombre d’hystéromètres** (IUDUtso) ________
Number of Tenaculum **Nombre de tenaculums**?(IUDTen) ________
Number of Gynecologic table **Nombre de tables gynécologiques** (IUDGyn) ________

- - Today does this clinic have materials and antiseptics for IUD insertion (IUDantisept)?

**Aujourd’hui le centre de santé a-t-il les matériaux et l’antiseptique pour l’insertion des DIUs** Yes (1) No (2)

- - Today does this clinic have sterile gloves (IUDglov)?

**Aujourd’hui le centre de santé a-t-il les gants stériles** ? Yes (1) No (2)

Number of Lamps for viewing the cervix (lampcervix)

**Nombres des lampes** **visualiser le col de l'utérus** ________

1. How many items for insertion of implant (Jadelle) do you have in your clinic? **Votre centre de santé a-t-il combine des items pour l’insertion des implants?**

Number of Implants **Nombre d’implants** (IMPmeth) _______

Number of Disposable Jadelle insertion kits **Nombre de kits jetables de Jadelle** ?(IMPKitDisp) ________

Number of NON-Disposable Jadelle insertion kits

**Nombre de kits non-jetables de Jadelle?** (IMPKitNonDisp) ________

Number of Lamp Halogen **Nombre de lampes halogènes**? (Halolight) ________

Number of Scalpel/blade **Nombre de scalpel ou lame** (IMPscalp) ________

- - Does the clinic have local anesthetic (needle, syringe, lidocaine/ligNOcaine) (IMPAna)

**Le centre de santé a-t-il l’anesthésique local ?** Yes (1) No (2)

- - Today does this clinic have materials and antiseptic to clean the arm (gauze, betadine) (IMPantisept) **Aujourd’hui le centre de santé a-t-il les matériaux et l’antiseptique pour nettoyer le bras ?** Yes (1) No (2)
  - Today does this clinic has bandage for the arm (IMPband) **Aujourd’hui le centre de santé a-t-il le pansement pour le bras?** Yes (1) No (2)

1. Do you currently use any promotional materials for the IUD or implant? (LARCProm) **Le centre de santé utilise-il actuellement du matériel promotionnel pour le DIU ou Implant ?** Yes (1) No (2)
   Please specify (LARCpromspec) **Veuillez spécifier**: ______________________________________________________________________________________________________________________________

______________________________________________________________________________________________________________________________

______________________________________________________________________________________________________________________________

______________________________________________________________________________________________________________________________

______________________________________________________________________________________________________________________________

1. Can you think of any other logistical barriers that need to be addressed to introduce or expand the number of LARC clients at your clinic? (LARCLOGBAR) **Pouvez-vous penser à d'autres obstacles logistiques qui doivent être abordées d'introduire ou d'augmenter le nombre de clients des méthodes de longues durées à votre centre de santé ?**

Please specify: **Veuillez specifier** :______________________________________________________________________________________

______________________________________________________________________________________________________________________________

______________________________________________________________________________________________________________________________

______________________________________________________________________________________________________________________________

______________________________________________________________________________________________________________________________

1. PSF is developing a couples’ family planning program with a focus on IUD/Implant. Would it be possible to recruit couples from your infant vaccination and family planning services for this program? (CFPCINFVAC)

**PSF est entrains d’élaborer un programme de planification familiale pour les couples qui se focalise sur les DIU / implants. Serait-il possible de recruter des couples des services de vaccination infantile et planification familiale pour ce programme ?** Yes (1) No (2)

1. What would be some obstacles that you may face in implementing this program (CFPCBAR)? **Quels seraient les obstacles que vous pourriez rencontrer dans la mise en œuvre de ce programme ?**

Please specify: **Veuillez spécifier :** _____________________________________________________________________________________

______________________________________________________________________________________________________________________________

______________________________________________________________________________________________________________________________

______________________________________________________________________________________________________________________________

______________________________________________________________________________________________________________________________

1. Do you have any partners/funders that help you provide family planning services? For example: MOH, FHI 360, JHPIEGO, etc. (FPPartner)? **Avez-vous des partenaires ou bailleurs de fonds qui vous aident à fournir les services de PF?**

Please specify: **Veuillez specifier**:_______________________________________________________________________________________

______________________________________________________________________________________________________________________________

______________________________________________________________________________________________________________________________

______________________________________________________________________________________________________________________________

______________________________________________________________________________________________________________________________

1. Do you have any questions about what we have discussed or any suggestions for us? (IIQs) . **Avez-vous des questions ou des suggestions sur ce que nous avons discuté ?**

Please specify: **Veuillez spécifier** ______________________________________________________________________________________

______________________________________________________________________________________________________________________________

______________________________________________________________________________________________________________________________

______________________________________________________________________________________________________________________________

______________________________________________________________________________________________________________________________
